# Occupancy of Urban Habitats by the Jersey Tiger Moth Is Revealed by Social Media Data but Not Traditional Monitoring

**DOI:** 10.1002/ece3.71086

**Published:** 2025-03-13

**Authors:** Nile Stephenson, Nathalie Pettorelli, Regan Early

**Affiliations:** ^1^ Department of Zoology University of Cambridge Cambridge UK; ^2^ University Museum of Zoology Downing Place, Cambridge Cambridge UK; ^3^ Centre for Ecology and Conservation Penryn Campus, University of Exeter Cornwall UK; ^4^ Institute of Zoology Zoological Society of London London UK

**Keywords:** biological records, climate change, *Euplagia quadripuncteria*, range shift, social media

## Abstract

As the world's climate changes, species are undergoing range shifts. Range shifts are generally documented using databases such as the Global Biodiversity Information Facility (GBIF), which largely contain data from monitoring schemes and wildlife surveys. Such databases have two major limitations: (i) data may be spatially biased because traditionally surveyed areas are in rural habitats and (ii) there is a time lag between formal monitoring and survey data collection and assimilation into GBIF, which means rapid range shifts cannot be tracked. Alternative data sources, such as social media, could provide information on species distributions and range shifts that compensate for spatial biases in GBIF records because social media data may be collected outside traditionally surveyed areas. Such data are also usually shared online immediately after a wildlife sighting. The complementarity of GBIF and social media data, however, has rarely been assessed, particularly when tracking range shifts. Despite their potential utility, social media data may be particularly prone to temporary trends or geographic variation in behaviour that are not understood. We lack tools with which to counter these biases. To address these knowledge gaps, we compare the habitat usage revealed by biological records of the Jersey tiger moth from GBIF and from social media data sources (Instagram and Flickr). We develop a novel method to investigate recorder bias in social media data and compare between data sources. We find that biological records from Instagram reveal greater than expected occurrence in urban environments. Recorder effort differs notably between data sources and Instagram complements GBIF by recording species in areas unaccounted for by GBIF. By incorporating recorder effort metrics, data from social media sources could be used to improve monitoring of range‐shifting species in urban spaces.

## Graphical abstract

Photos posted on social media could provide information on species' responses to climate change that is up to date and from areas under‐represented in traditional biological record data. Instagram and iNaturalist data reveal that the Jersey tiger moth, a species that is shifting northwards in Europe, is using urban habitat. This phenomenon is greatly under‐predicted by more traditional biological record data. We provide a method to account for the considerable spatial and temporal variation in social media data and recommend that such data be used to complement traditional data when tracking fast‐moving range‐shifts.

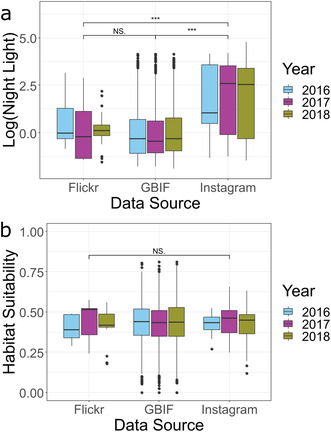

## 1 Introduction

Species around the globe are redistributing in response to anthropogenic climate change (Hamann and Wang 2006; Dennis et al. 1999; Van Der Putten et al. 2010). Range‐shifting species elicit positive (Dawson et al. 2011) and negative (Pettorelli et al. 2019; Wallingford et al. 2020) ecological and societal impacts (Cranston et al. 2022); thus, there is a need to track range shifts. Tracking range shifts requires large, high‐quality occurrence datasets, such as those provided by online databases like the Global Biodiversity Information Facility (GBIF) (Hirzel et al. 2001; The Global Biodiversity Information Facility 2020). GBIF collates occurrence data from a range of sources, which historically were from scientific surveys, though dedicated citizen science schemes are now extremely common (Anderson et al. 2016; Petersen et al. 2021). The vast majority of scientific surveys occur in a species' ‘natural’ habitat—where a species is historically likely to be found—which may bias occurrence records from databases such as GBIF towards rural locations. However, recent studies report that urban environments are important to range‐shifting species; many range shifters have been found to be human‐associated, often occurring in gardens or unintentionally transported into cities as passengers on trade vessels (Van Der Veken et al. 2008; Estrada et al. 2018). Therefore, the possibility of relatively urban environments being under‐represented in databases such as GBIF may cause a gap within occurrence data records for range shifters. Monitoring arrivals in human‐dominated landscapes such as urbanised areas may therefore reduce spatial bias in predictive models and inform the association between range‐shifting species and urban habitats.

Another challenge for sourcing data on range shifts is that there may be a time lag (up to 3 years) associated with the process of recording, verification and agglomeration of traditional scientific surveys and occurrence data becoming available, for example, through GBIF (Samy et al. 2013; Kusber et al. 2009). However, the speed and magnitude of range shifts necessitate more rapid data availability (Straub et al. 2016; Sinka et al. 2020).

One potential solution could be the implementation of community science projects, which have been shown to produce high‐quality occurrence data quickly (Delaney et al. 2008; Maistrello et al. 2016; Sumner et al. 2019; Millard et al. 2021), though these can generate spatial biases (e.g. Daru and Rodriguez 2023). However, community science projects often require vast resource expenditure and many willing participants (Sumner et al. 2019). Another potential avenue to gather occurrence data quickly within a variety of environments is via social media (Jarić et al. 2020; O'Neill et al. 2023). Social media users may upload georeferenced photographs of a species of interest incidentally (Jarić et al. 2020). Photos of a focal species are often uploaded to social media immediately, expediting the process of gathering data. Such data have been shown to improve systematic conservation planning (Chowdhury, Fuller, et al. 2024) and assist in predicting species' habitat suitability (O'Neill et al. 2023).

Furthermore, because the majority of humans reside in urban environments and urban environments benefit from a good internet connection, it is likely that social media will survey these environments. Social media sources may reveal the use of urban habitat overlooked within traditional surveying methods that target rural areas (Hall et al. 2017).

Despite the advantages above, social media data could also be patchy and prone to a higher degree of spatial recorder bias than traditional ecological data. Heterogeneous recorder effort can cause over‐ and under‐estimation of the suitability of particular environmental conditions in Habitat Suitability Models (HSMs). Patchiness could be due to hotspots of social media use within highly urbanized areas, and users may be heavily influenced by trends, leading to a period of intense interest in a small number of species (Mancini et al. 2018). It is therefore particularly important to understand the role of spatial and temporal recorder effort bias in social media data. There may also be variations in spatial bias and the influence of trends between different social media platforms, so we need to understand how recorder effort differs between platforms.

In this study, we compare the information content provided by different sources of occurrence data of a range‐shifting species, the Jersey tiger moth (JTM), *Euplagia quadripuncteria* (formerly *Callimorpha quadripuncteria*). JTM is a day‐flying, recognizable, abundant lepidopteran currently undergoing rapid range shifts due to climate change (Waring and Townsend 2017). JTM is a generalist species, likely to be able to make use of urban environments (Sorace and Gustin 2009), and is also visually striking; therefore, potentially generating interest on social media platforms. We: (i) model annual habitat suitability for JTM in a portion of Europe during a period of changing climate (2000–2018) using data from GBIF; assess whether occurrences of JTM from social media data sources (Twitter, Flickr and Instagram) are found (ii) in areas with different habitat suitability than that calculated with GBIF models; or (iii) in more urban areas, and (iv) investigate how recorder effort affects JTM occurrence across all data sources. We predict that: (i) occurrence data from social media platforms are found in areas that models based on GBIF data would predict to be of low habitat suitability or that are more urban; and (ii) accounting for recorder effort will be particularly important for the modeling of species distribution using social media data.

## 2 Materials and Methods

### 2.1 Data Collection

Occurrence data were collected from four sources: GBIF, Instagram and Flickr. Instagram and Flickr were selected because biological records could be extracted with relative ease. We considered including occurrence data from Twitter and Facebook but were unable to find enough geolocated, verifiable images of JTM during our study period. Records from each source were collected from between 2000 and 2018, as these were the years when comparable environmental data could be gathered and where JTM had been sufficiently sampled (> 50 occurrences per year) from GBIF across the selected study region.

**FIGURE 1:**
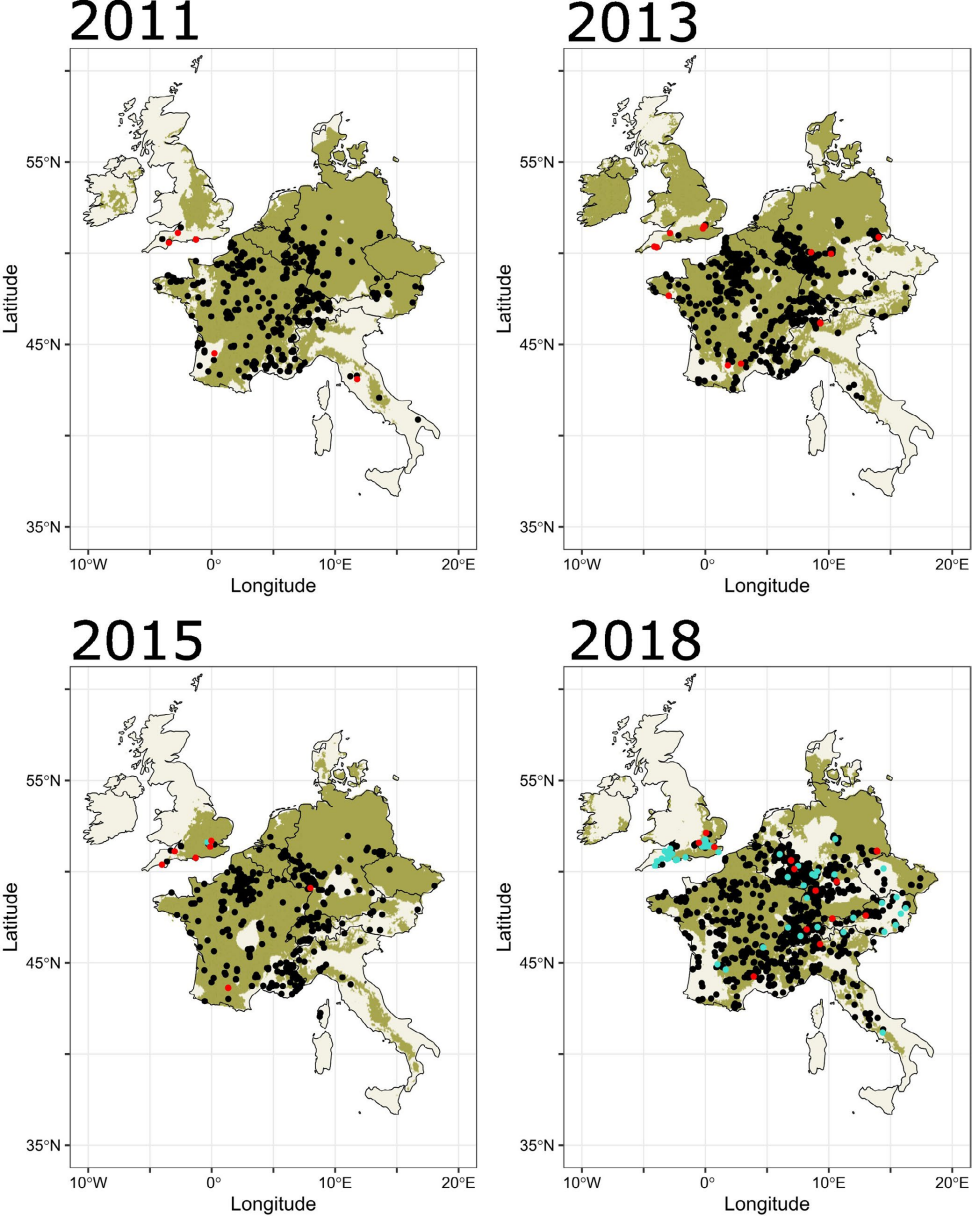
HSMs for JTM across the study region. Green areas represent suitable habitat for JTM (sensitivity = 0.9); black points are from GBIF; red points originate from Flickr and turquoise from Instagram. Maps presented here selected to display general pattern of changes in habitat suitability (all other maps see Figure S5). Habitat suitability was derived from information on maximum temperature, coefficient of variation in maximum temperature, total precipitation and coefficient of variation in total precipitation using bioClim models produced with the *dismo* package in R.

The study region included the UK, Republic of Ireland, France, Belgium, the Netherlands, Luxembourg, Switzerland, Czech Republic, Austria, Germany, Denmark and Italy (Figure 1). This region represents a large proportion of the known distribution of JTM and includes nations from which biological records were reported to GBIF throughout 2000–2018. Although the region does not encompass the hottest component of the species' climate niche (as records in this region were too sparse), this should not affect predictions of habitat suitability for the range shift of JTM at the northern edge of its range, where conditions are cooler.

Search terms (Table 1) were applied for Instagram and Flickr to both original posts and their subsequent comments to account for individuals who were unable to identify JTM and were seeking identification. We only used occurrences derived from posts and tweets that included an image of adult JTM. Duplicates from social media data arising from people sharing the same information on different platforms were removed. These were identified by identical date and coordinates and visual inspection for the same individual recorded in multiple photographs. We retained duplicates in the dataset for the data source in which they first occurred. All occurrence data derived from social media platforms were manually checked to ensure that identification of adult JTM was correct. Only occurrences that fell within months of the year when JTM adults fly (July–September (Waring and Townsend 2017), when 95% of the GBIF non‐larval sightings were recorded) were retained in the instances of Flickr and Instagram. All larval records were removed from our GBIF dataset. We only obtained 16 records from Twitter, so we did not include this data source in any further analyses. For data from Flickr, georeferences were automatically extracted using a custom script, but for Instagram, georeferenced data was manually collected from individual posts where such information was provided. Where georeferences were absent, no data were collected.

**TABLE 1:** Summary of the search terms and processes used to collect biological records of JTM across the study region. Hits refer to the quantity of successful occurrences that contained all of the required information for the study within the time span of the study (2000–2018) and within the study region (the UK, Republic of Ireland, France, Belgium, the Netherlands, Luxembourg, Switzerland, Czech Republic, Austria, Germany, Denmark and Italy). Note that searches on Instagram are limited to hashtags rather than caption text. Data are available from https://figshare.com/s/94529defd9aa93d18426, except GBIF data, which are available from the link in the table. Note that subsequent checking for common names in French and Dutch revealed only two additional georeferenced Flickr or Instagram records that we did not include in analyses.

Data source	Search terms(s)	Process	Hits
GBIF	*Euplagia quadripunctaria* *Callimorpha quadripunctaria*	Downloaded from GBIF at https://doi.org/10.15468/dl.dtfjkv (GBIF.org 2020)	2891
iNaturalist	*Euplagia quadripuncteria* *Callimorpha quadripunctaria*	Downloaded from GBIF—iNaturalist records were separated from GBIF records	1057
Flickr	*#Euplagia quadripuncteria* #Jersey Tiger #Russischer Bär #Spanische Flagge #La falena dell'edera #Přástevník kostivalový #Rhodos‐Bjørn	API query using python code; gather geographical data using *FlickrAPI* package in R (Mair and Ruete 2016)	106
Instagram	#Euplagiaquadripuncteria #Jerseytiger #RussischerBär #SpanischeFlagge	Manual search	134

We also performed a supplementary analysis on a subset of GBIF data—iNaturalist records. iNaturalist is typical of an increasing number of citizen science schemes (also including observation.org, Naturgucker, Artenfinder) where individuals upload photos of their observations, which are then verified by other volunteers before being uploaded to GBIF. Verification is often near‐instant, and uploads occur regularly, so such data might be particularly useful in tracking range‐shifting species. Citizen science schemes contribute an increasing amount of data to GBIF, particularly in data‐poor countries (Amano et al. 2016). Such schemes may offer a possible middle ground between social media and scientific surveys, possibly overcoming any rural bias in scientific surveys, while still being collected and verified by those dedicated to formal biological recording. Therefore, we performed a supplementary analysis separating iNaturalist from GBIF records and repeated all subsequent analyses with iNaturalist included as an additional data source.

To represent JTM's climatic niche, we used four climatic variables: average maximum temperature, coefficient of variation in average maximum temperature, total precipitation and coefficient of variation in total precipitation. These four variables have been found to be dominant factors in the range shift and migration of other lepidoptera (Sparks et al. 2007, 2005). Climatic data were all calculated per year for the flying time of JTM. Climatic data were gathered from WorldClim at a 2.5 min spatial resolution (~21 km^2^) (WorldClim 2020; Harris et al. 2014). We note that the CHELSA dataset is an improvement on WorldClim, but the differences are very small within Europe and within the non‐mountainous habitat that JTM largely occupies (Karger et al. 2017). Our goal was to understand the impact of social media data and recorder effort on biomonitoring of range shifts rather than make the most accurate range‐shift prediction possible, and it is highly unlikely the small, unsystematic differences between CHELSA and WorldClim in our study region would affect these results. Other climatic layers were not included to avoid overfitting HSMs, underpredicting potential distributions and tolerances under climatic conditions where species may be underreported (Early and Sax 2014).

We used night light to capture the degree of urbanisation within the areas predicted suitable by the climatic HSM (Gaston et al. 2015). Night light data were collected from December of every year (data from summer months may not be an accurate representation due to the lighter summers in the northernmost parts of the study region). Data were collected from the National Centers for Environmental Information (National Centers for Environmental Information 2019) and converted to a 2.5 min spatial resolution (~21 km^2^) by averaging. Stray light, lightning, lunar illumination, and cloud cover are all removed from the average measure of illumination prior to the calculation of averages for each layer. Only data from 2012 onwards were comparable between years, so for all years prior to 2012, the night light dataset from 2012 was used.

### 2.2 Calculating Recorder Effort for Data Sources

Accurate estimations of recorder effort have been a significant quandary for many previous studies (Dennis et al. 1999; Isaac and Pocock 2015; Hassall and Thompson 2010; Casey 2016). Here, we defined recorder effort as a ratio between the number of records of a species in a location and the species' estimated abundance in that location. High ratios indicate grid‐cells where a species is detected frequently relative to its abundance, and thus recorder effort is high. Recorder effort could not be calculated for JTM itself since there are no independent estimates of its abundance across the study region. Abundance data for other insects with which to calculate recorder effort are also rarely available. Therefore, we used a surrogate species: the Eurasian blackbird, *Turdus merula* , which has a consistent range and abundance between 2000 and 2018, is easily identifiable, is charismatic (thus of interest to social media users), and has been recorded across all data sources considered between 2000 and 2018 across the study region. Furthermore, the blackbird occupied both urban and rural environments, so using blackbirds to estimate recorder effort should minimise the difference in abundance recording between urban and rural areas. We therefore judged records for this species' occurrence to reflect the interest in recording wildlife in a given time or location (BirdLife International 2016). Blackbird abundance data seem likely to allow for comparison of recorder effort between localities and time periods that could be applied to a wide range of taxa. Estimations of blackbird abundance throughout Europe were acquired from the European Breeding Bird Atlas 2 (Keller et al. 2020).

In order to calculate the recorder effort ratio for each data source, we collected blackbird occurrences using the search terms and processes in Table S1. A ratio between the number of blackbird records for each data source and the estimated abundance was calculated for each UTM grid cell (Figures S1–S3) from the European Breeding Bird Atlas (~50 km^2^ resolution, although some cells varied in size). Recorder effort for GBIF was calculated for all years, whereas the recorder effort for other sources was produced for 2016, 2017 and 2018 (the years for which social media data sources were studied). Other approaches to recorder effort have used the number of species recorded in an area (Isaac and Pocock 2015), our approach has the advantage that it is not affected by species richness. Moreover, if social media users are indeed more likely to record eye‐catching or charismatic species, their recorder effort may not be reflected by the overall number of species recorded in a given time or location.

There is a potential confound within this measure of recorder effort given that traditional data are used to estimate blackbird relative abundance: blackbird abundance may be underestimated in urban environments as per our own hypotheses. Using blackbird abundance as the denominator in recorder effort calculations could mean we overestimate recorder effort in urban areas, relative to rural areas. However, this should not affect the relative difference in recorder effort between data sources within urban areas.

### 2.3 Comparing JTM's Habitat Usage Obtained From Different Data Sources

In order to ask whether social media data sources included more urban records than GBIF did we compared the logged intensity of night light between records from each source.

To ask whether GBIF data underestimated the urban component of JTM's range shift, we compared social media records to habitat suitability calculated using GBIF records. GBIF HSMs were produced using bioclim in the *dismo* package. Bioclim is a distance‐based, boxcar method for assessing habitat suitability based on the similarity of bioclimatic variables between points in space (Beaumont et al. 2016). Thus, bioclim is simple and robust, which is ideal for comparing habitat suitability at points in different regions and time periods, when the placement of pseudo‐absences might strongly affect habitat suitability estimates. First, a single historic (‘GBIF‐calculated’) HSM was calculated for 2000–2009 using the average climatic variables and occurrences of JTM in GBIF from these years. This model was a suitable baseline as it would average out any unusual bioclimatic conditions that could occur within a single year and boasted a relatively large sample size (*N* = 1502). We used a randomly selected 80% of the data points as training data to construct the historic model. Model performance (measured as area under the receiver operating curve; AUC, and calculation of the Boyce Index (Roy et al. 2016)) was calculated using the remaining 20% of the data as a testing dataset. In order to calculate the AUC and Boyce Index, pseudo‐absences were generated by selecting random points from the same study region as the presence data with a 50% prevalence. When predicting suitable and unsuitable habitat, we used a sensitivity threshold of 0.9. This maximized the potential suitable habitat for JTM and partially accounted for underreporting. A threshold of 0.95 was also attempted but discarded since it classified areas that are almost certainly unsuitable for JTM (such as the Scottish Highlands [Waring and Townsend 2017]) as suitable.

The historic model was then used to predict the relative habitat suitability for JTM for each year between 2010 and 2018 across the study region using the climatic variables for each year. We extracted habitat suitability from HSMs at the coordinates of each occurrence of JTM from each data source across the study region for the years 2016–2018. The years 2016–2018 were selected as these had relatively large sample sizes for all data sources. In order to test if different data sources recorded JTM in areas of differing habitat suitability across the study region, a linear model was constructed with predicted habitat suitability at each occurrence of JTM as the response variable and the source of the occurrence data as a predictor variable. Any differences between sources were then investigated via Tukey's post hoc test. Predicted habitat suitability data extracted from JTM occurrence locations were log transformed to homogenise the variance and meet assumptions of linearity. Following this, to investigate if any differences were due to urbanisation, a linear model was produced with night light extracted from JTM occurrence locations as the response variable and the source of the occurrence data as the predictor variable. Night light data were square root transformed to meet the assumptions of linearity. Any differences between sources were then investigated via Tukey's post hoc test.

Any geographical area with extremes of climate could generate a bias when testing between predicted habitat suitability if one data source happened to be overrepresented in this extreme. For example, if Flickr was overrepresented in Italy and Italy was predicted to have a low habitat suitability for JTM (based on data from GBIF) due to extreme temperature, then this could confound a result that suggested that data from Flickr were located in areas of significantly lower habitat suitability. Since Italy represented the hottest parts of JTM's range in the study area, we repeated all the above analyses without Italy included in the models and then compared the output of both Italy‐included and Italy‐omitted analyses. We did not do this for the coldest part of the range of JTM since the range shift into these colder climates (e.g., the UK) is foundational to our questions.

### 2.4 Assessing the Contribution of Recorder Effort to the Occurrence of JTM

In order to assess whether recorder effort affected the distribution of known occurrences of JTM throughout the study region, three generalised linear mixed models (GLMMs; one for each data source) were constructed. GLMMs were constructed for the years 2016–2018, with the presence/pseudo‐absence of JTM throughout the study region as a binary response variable. Recorder effort, habitat suitability from the historic model, and the interaction between the two were predictor variables in each model. Year was included as a random effect to account for a lack of independence between years. As above, AIC selection was then implemented to select the best model. Habitat suitability and recorder effort were both standardised by subtracting their means and dividing by their standard deviations. GLMMs were constructed using the *glmmTMB* package and had a binary error structure and a logit link function. All analyses were conducted in R version 4.0.0 (R Core Team 2020). Code is available from https://github.com/nis38/JTM.

## 3 Results

**FIGURE 2:**
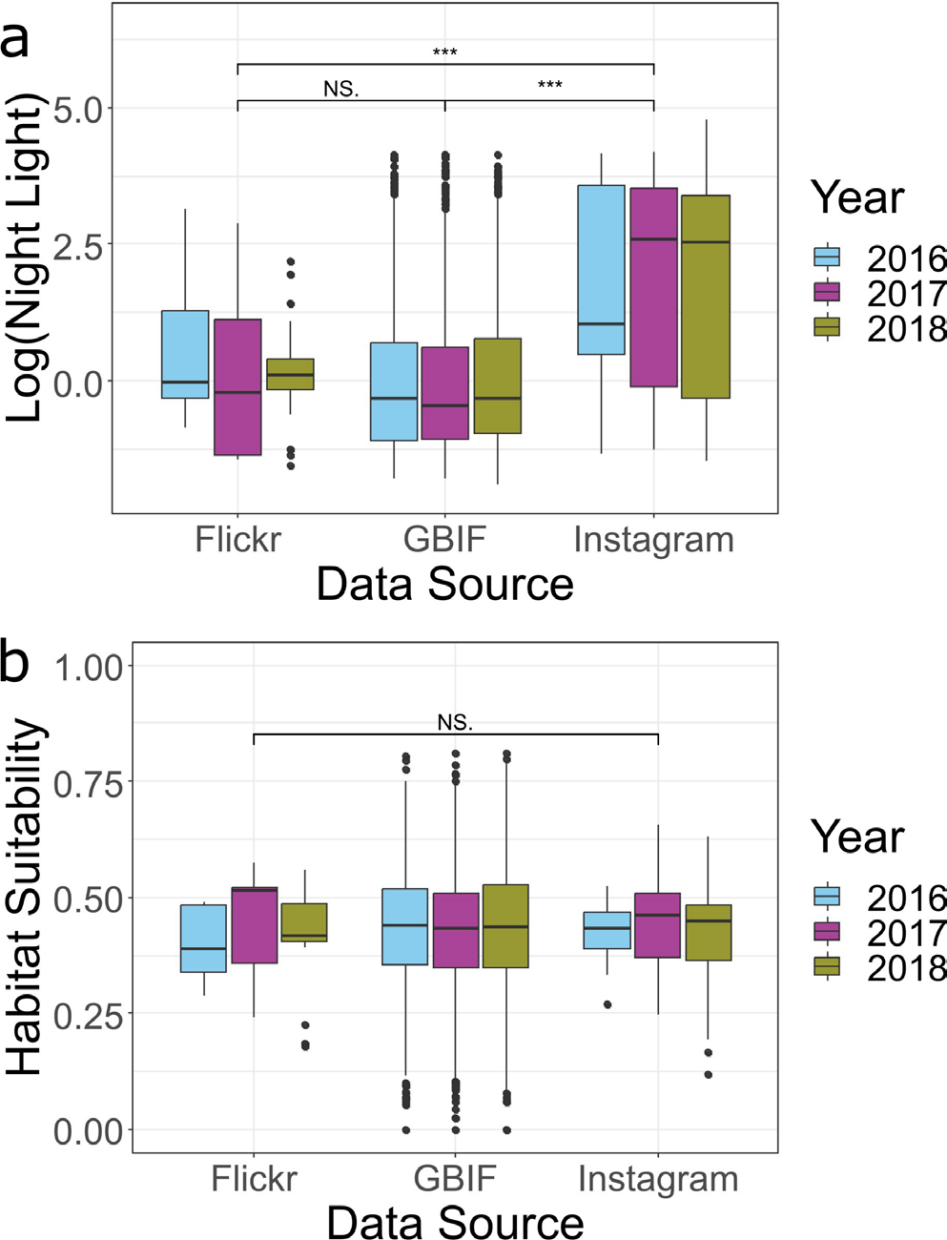
Differences in (a) night light (urbanisation) and (b) habitat suitability between different sources of occurrence data. Data were taken from the selected study region across 2016–2018. (a and b): Horizontal black bars denote median; vertical bars denote quantiles; NS denotes no significant difference, asterisks denote statistically different variables, and quantity of asterisks denote size of *p*‐value (***p < 0.001).

The intensity of night light varied significantly between records from 2016 to 2018 from each data source (ANOVA; *F*_2,3060_ = 111.8, *p* < 0.001, Figure 2a), but GBIF‐calculated habitat suitability did not (ANOVA; *F*_2,3063_ = 0.04, *p* = 0.961, Figure 2b). At GBIF and Flickr occurrences, urbanisation (night light) was significantly lower than at Instagram occurrences (Figure 2a; Table 2). When Italy was removed from the study area, the relationships between data sources and urbanisation (night light) remained the same, but occurrences from Instagram had significantly lower GBIF‐calculated habitat suitability than occurrences from GBIF (Figure S4).

**TABLE 2:** Summary of Tukey's post hoc tests between (log) habitat suitability and urbanization (square root night light) for three sources of JTM occurrence data.

Comparison	Habitat suitability difference	Habitat suitability *p*‐value	Urbanisation difference	Urbanisation *p*‐value
GBIF–Flickr	−0.005	0.965	−0.263	0.362
Instagram–Flickr	0.003	0.988	1.554	**< 0.001**
Instagram–GBIF	−0.002	0.991	1.817	**< 0.001**

*Note:* Bold *p*‐values indicate significance at *p* < 0.05.

When iNaturalist was separated from GBIF occurrence data, we found that there were significant differences in habitat suitability between iNaturalist and Flickr, iNaturalist and GBIF and iNaturalist and Instagram (ANOVA; *F*_3,3886_ = 33.84, *p* < 0.001, Figure S7a, Table S2), and that occurrences of JTM iNaturalist were found in areas of significantly higher urbanisation than those from the remaining GBIF records, but significantly and substantially less urbanisation than JTM occurrences from Instagram (ANOVA; *F*_3,3881_ = 77.94, *p* < 0.001, Figure S7b, Table S2). We found that occurrence records from iNaturalist had a positive association with GBIF‐calculated habitat suitability and recorder effort (Figure S8; Tables S3 and S4).

**FIGURE 3:**
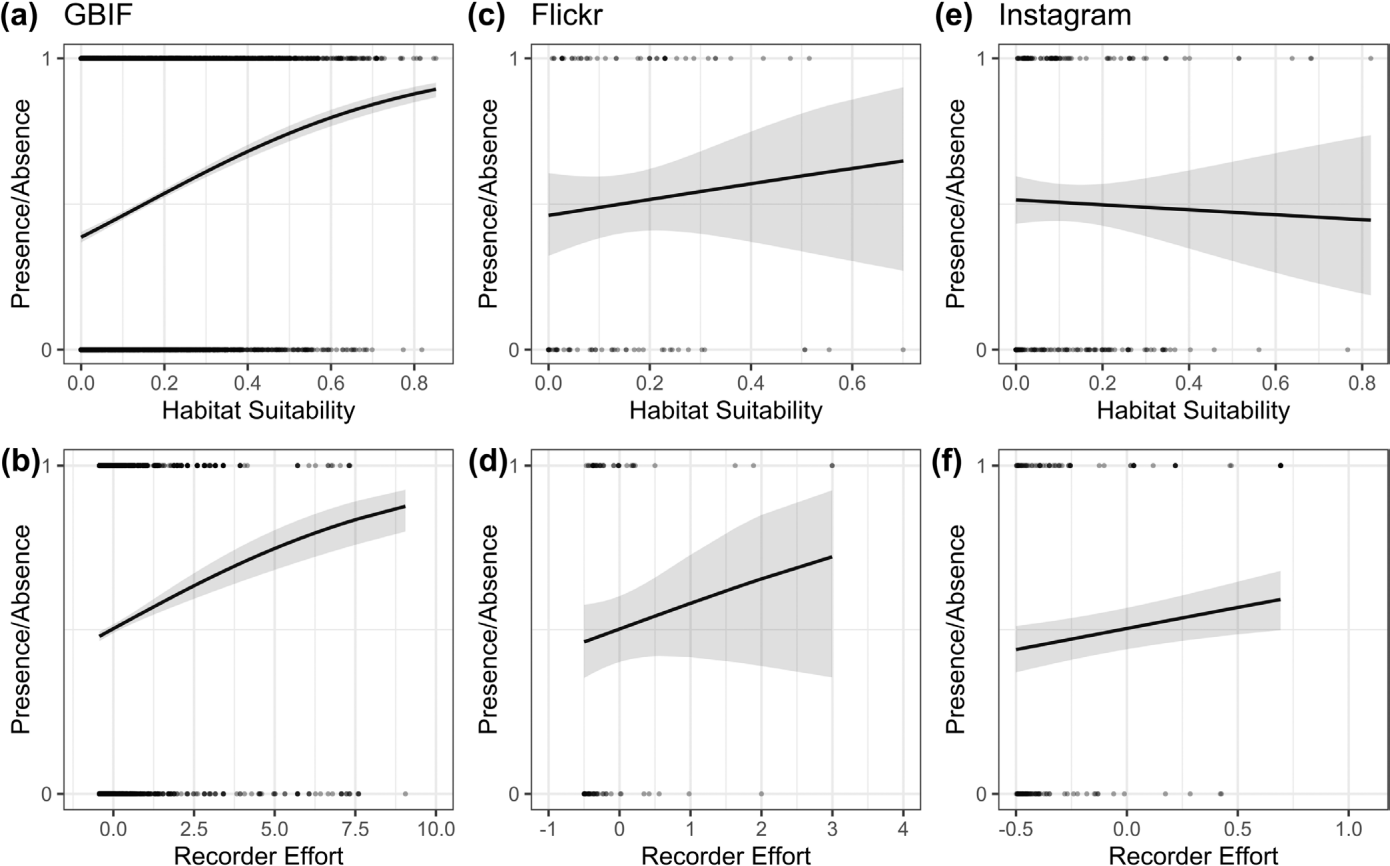
Effect of standardised values of habitat suitability (a, c, e) and recorder effort (b, d, f) on the presence of JTM for GBIF data (a and b), Flickr (c and d) and Instagram (e and f). 0 on the *y*‐axis refers to a pseudo‐absence, 1 refers to the presence of JTM. Lines are predicted from GLMMs with year as a random effect; however, due to a low variance explained by year (mean standard deviation = 0.020), only the average effect across years was plotted per panel. The grey area denotes the 95% confidence interval.

Recorder effort and GBIF‐calculated habitat suitability affected the 2016–2018 occurrences of JTM in all data sources (Figure 3, Table S4). Unsurprisingly, occurrence records from GBIF were more likely to be present in areas of high predicted habitat suitability, but GBIF also mostly recorded JTM where recorder effort was high (Figure 3a,b, respectively). Occurrence records from Flickr were also associated with areas of relatively high recorder effort and were associated with areas of high GBIF‐calculated habitat suitability (Figure 3c,d, respectively). Occurrence records from Instagram were not associated with GBIF‐calculated habitat suitability but had a positive relationship with recorder effort (Figure 3e,f, respectively).

Even though the geographic background for the HSMs did not include the species' entire range, this did not affect our results, since all post‐2009 JTM records are found within climate conditions that are analogous to the historical range (Figure S6). GBIF‐calculated suitable habitat across the study region was relatively consistent between years, with notable exceptions in the UK, Republic of Ireland, Italy and Denmark (Figure 1 and Figure S6). The mean (±standard deviation) AUC of HSMs was 0.649 (±0.043) and the mean (±standard deviation) Boyce Index was 0.523 (±0.441).

## 4 Discussion

In this study, we demonstrate that occurrence records from Instagram are found in different and more urbanised locations compared to occurrences from traditional datasets, such as GBIF. The similarity in habitat suitability between data sources indicates that Instagram, Flickr and iNaturalist records are not found in climatically dissimilar areas from GBIF records across the study region. However, unlike Flickr, Instagram records were not more likely to be found in areas of higher habitat suitability. Flickr data appeared to be least affected by recorder effort. However, contrary to our predictions, occurrence data from Flickr offer a somewhat similar outlook to that provided by GBIF records. There was a notable difference in the environments surveyed by different social media platforms, with Flickr data occurring in more rural locations than data from Instagram, which occurred in more urbanised (significantly higher night light) areas. We therefore highlight the utility of these social media platforms as additional and complementary sources of data to traditional databases, such as GBIF.

As predicted, the majority of post‐2009 occurrence records from GBIF for JTM within our study region fell within more rural areas. Contrary to this, as predicted, Instagram largely contains data from highly urban zones, likely because urban areas are densely populated by humans and have good internet connections, leading to geographic trends in human behavior affecting whether they upload data to social media. However, data from Flickr are more rural than data from Instagram. Flickr is tailored towards individuals with an interest in the quality of photography, which may attract wildlife photographers intent on capturing wildlife in relatively rural environments rather than in urban zones. iNaturalist, which collects citizen science data more formally than social media, also appears to under‐record JTM presence in urban zones. This suggests that the recent growth in citizen science data within GBIF may still under‐record urban areas. Overall, this demonstrates the utility of social media sites such as Instagram to fill a void in the occurrence records provided by traditionally used data sources such as GBIF. On the flip side, Instagram data are likely to under‐estimate the rural component of species' ranges. Although we only studied one species, we think it is likely that the urban–rural differences between databases would remain for similar (colourful, eye‐catching) species that would likely be uploaded to social media. An additional utility of both Flickr and Instagram is that they may make species records publicly available more rapidly than GBIF.

Our results also highlight the importance of accounting for recorder effort. The positive effect of recorder effort on GBIF (very strong) and Flickr (less strong, with wide confidence intervals) occurrences indicates that JTM is detected where recorders are searching for wildlife (as indicated by blackbird recording effort). Thus, GBIF's and Flickr's predictions of low JTM occurrence in more urban areas (Figure 2a) are not necessarily trustworthy. There was also a positive relationship between the location of JTM records from Instagram and recorder effort. This suggests that Instagram is also detecting species in areas where wildlife is being recorded by the majority of its users (as indicated by blackbird recording effort). However, Instagram records, while in areas of (GBIF‐calculated) suitable habitat, are detected in areas of significantly higher urbanisation (night light) than GBIF or Flickr, indicating that the recorder effort of GBIF is focused in more rural areas, whereas the recorder effort of Instagram is focused in more urban areas.

It should be noted that recorder effort was particularly geographically uneven for social media sources, and our results could be affected by this patchiness. In addition to what we detected, there may also be a novelty bias towards range‐shifting species, inflating records when species first arrive in an area. Given the varying but broadly important effect of recorder effort, developing improved recorder effort metrics could be particularly important to the use of social media data in biogeography and range‐shift ecology.

Most existing metrics for estimating recorder effort from occurrence data alone rely on occurrence data or species lists for a suite of species that are thought to be similarly recorded (Hill 2012; Isaac et al. 2014). These metrics are data‐intensive and influenced heavily by the species selected (Isaac et al. 2014). It is highly likely that the difference in recorder practices between datasets would prevent the current form of these metrics from being used for valid comparisons between datasets. In contrast, the known abundance of a widespread and well‐noticed species (in this case blackbird) provides a single comparator for all datasets. We suggest this approach provides a good proxy to illustrate the relative unevenness of recording in different data sources. Nonetheless, it's possible that blackbird recorder effort does not reflect JTM recorder effort, particularly within GBIF, since surveys for different taxa are likely to employ differing sampling techniques and contributors (Mair and Ruete 2016). It would have been preferable to use the abundance of JTM itself, another day‐flying moth, or even another insect. Such data do not currently exist, and abundance data for less‐recorded taxa are unlikely to be available in the near future. We therefore recommend using one or more widespread and well‐recorded species, regardless of taxon, as a first step to understanding recording effort between areas, taxa, or time periods in social media data. However, we recommend closer evaluation of the congruence between recording effort for different taxa. In the longer term, we recommend investigating the application of methods designed for occurrence datasets, for example, Frescalo (Hill 2012), to social media data.

Range‐shifting and invasive species have previously been found to be human‐associated, persisting in urban parks and gardens (Van Der Veken et al. 2008). Although the extent of this association remains unknown, our results highlight the potential for social media data to track and understand range‐shifting species in urban zones. Since Instagram's focus is on photography, it could be used to track the arrival of eye‐catching or charismatic taxa in urban areas. However, a less recognizable or visually appealing species than JTM could generate fewer occurrences, and thus the repeatability of the use of Instagram data across different taxa requires further investigation. In addition, the collection of ad hoc social media data may present opportunities for researchers to assess wildlife management practices in urban and suburban areas. Surveys of bug hotels, bird feeders and mutualists from social media could be recorded to assess hotspots of positive management in cities, as well as areas that are deficient in their capacity to support biodiversity. Furthermore, social media data could be used to assess the persistence of endangered species in urban and suburban areas, adding to the work already compiled regarding the importance of gardens in supporting threatened or keystone taxa (Lowenstein and Minor 2016). Our study also suggests that there may even be scope for assessing the potential for urban spaces to propagate range‐shifts and invasions further in a similar way to forest corridors (Melles et al. 2011). It is clear that if robust and repeatable methodologies can be applied, social media data sources have a high potential to provide high‐quality data at speed. Furthermore, it is likely that these methods will only increase in importance as urbanization rises globally (Goddard et al. 2010). It is also noteworthy that social media platforms such as Twitter have been used to promote uptake of the UK ladybird survey, yielding insights into the spread of the Harlequin ladybird (Roy et al. 2016).

Scientific and policy‐maker interest in community science in urban areas is growing, given that urban environments are increasing, most people live in urban environments, and most nature experiences are close to home (Veerkamp et al. 2021). Noticing urban wildlife can improve mental and physical wellbeing (Aerts et al. 2018; Houlden et al. 2021), and increasing engagement with urban nature offers the opportunity for improved ecological literacy and nature connectedness, particularly amongst social groups that have historically had inequitable access to nature (Cooper et al. 2021; Amorim Maia et al. 2020). Our results further reinforce recent findings that social media platforms could be harnessed to assist in urban nature engagement and conservation (Persson et al. 2023; Langemeyer et al. 2018). While our results highlight a promising avenue for future studies and offer novel sources of data with new information, a fundamental area of improvement is the establishment of a rigorous and consistent methodology (Jarić et al. 2020). For example, recently, a standardised method for extracting species distribution records from Facebook groups has been made available (Chowdhury, Fuller, et al. 2024; Chowdhury, Ahmed, et al. 2024). A source of uncertainty for our study is that the search terms and the access and use of APIs (Application Programming Interface used to extract data) could not be made consistent across all social media data sources. The process by which data are attained would be benefited by greater consistency; the main barrier here is the expense of using the API services supplied by Instagram and Twitter. Both services have recency constraints and query limits associated with the free‐to‐use APIs, and the cost of more expansive API usage was outside of the budget of this study, costing up to £2000 per month depending on the service used at the time at which this study was conducted. This could be overcome with additional studies highlighting the importance of access to these data for scientists, thus prompting social media companies to produce an API service that is accessible to scientists. Alternatively, machine learning programmes such as UI Path could provide a more affordable and consistent method to gather data from online sources (Sirisuriya 2015). Implementation of alternative methodologies and different focal species are likely to increase the utility of Twitter, which was omitted from analyses due to a low sample size, and could permit the use of other social media sources not considered here due to data accessibility, such as TikTok or Facebook.

A further potential issue with social media use is that there is not necessarily equal utilization of these sources throughout all nations, particularly in those outside of Europe and North America. Search terms should also be considered with caution. We have included the search terms that yielded the most occurrences of JTM. Although various common names are not always simple to incorporate (as was the case here, with German names such as ‘Spanish flag’ and ‘Russian bear’, which yielded countless non‐moth results when searched), this is certainly worthy of consideration. Social media data sources are also driven by trends, which may contribute to the varying usefulness of different sources over time as the popularity and novelty of range‐shifting species wax and wane. Such an effect seemed to be apparent with JTM, where the inclusion of the moth on postage stamps in the Channel Islands was associated with an increase in GBIF occurrences in 2012 and 2013 (which also illustrates that even GBIF is not resistant to trends, which could be due to growing input from citizen science websites like iNaturalist). Nonetheless, such trends could also be a potential advantage to social media data sources. In theory, researchers could highlight species of interest to the public, thus generating a trend around focal organisms that could be used to generate social media occurrence records. Such strategies could increase the use of social media to record biological phenomena.

The results presented here support the idea that the combined use of traditional (GBIF) and social media (particularly Instagram) data sources generates a more complete understanding of the habitat use of range‐shifting species. Our study suggests that traditional and social media biodiversity data can contain different but complementary information regarding the habitat usage of a range‐shifting species. While GBIF captures the rural range of JTM across the study region, Instagram demonstrated that JTM also occupies highly urbanised environments. We illustrate that social media data may be particularly prone to variation in recorder effort, and characterising this should be a research priority. We suggest that data from social media should be used to complement more traditional data sources when tracking range‐shifting species. However, incorporating social media data into biodiversity monitoring platforms like GBIF would take considerable time and effort (Soriano‐Redondo et al. 2024). In the short term, it might be more effective to use such data on a case‐by‐case basis to tackle specific questions related to urban areas or rapidly shifting species. Utilising occurrence records from social media could be particularly important given the human‐associated nature of some range shifters, which often occupy parks and gardens in urban zones as well as rural spaces.

## Author Contributions

**Nile Stephenson:** conceptualization (equal), data curation (lead), formal analysis (lead), investigation (lead), methodology (lead), visualization (lead), writing – original draft (lead). **Nathalie Pettorelli:** conceptualization (supporting), investigation (supporting), methodology (supporting), supervision (supporting), writing – review and editing (supporting). **Regan Early:** conceptualization (equal), investigation (supporting), methodology (supporting), supervision (lead), writing – review and editing (lead).

## Conflicts of Interest

The authors declare no conflicts of interest.

## Supporting information


Data S1.

## Data Availability

The code and data used to run analyses is available at https://doi.org/10.5281/zenodo.14894013. The GBIF dataset on *Euplagia quadripuncteria* we used can be downloaded from https://doi.org/10.15468/dl.dtfjkv (GBIF.org 2020). The GBIF dataset on *Turdus merula* we used can be downloaded from https://doi.org/10.15468/dl.dn3vez. Other data we used are available at https://figshare.com/s/94529defd9aa93d18426.
