# Supplementary material for: Occupancy of Urban Habitats by the Jersey Tiger Moth Is Revealed by Social Media Data but Not Traditional Monitoring
Source: Ecol Evol. 2025 Mar 13;15(3):e71086. doi: 10.1002/ece3.71086 (PMC11904309; doi:10.1002/ece3.71086)
Supplement: Supplementary file 1 — Data S1. [file ECE3-15-e71086-s001.docx]

**Supplementary materials**


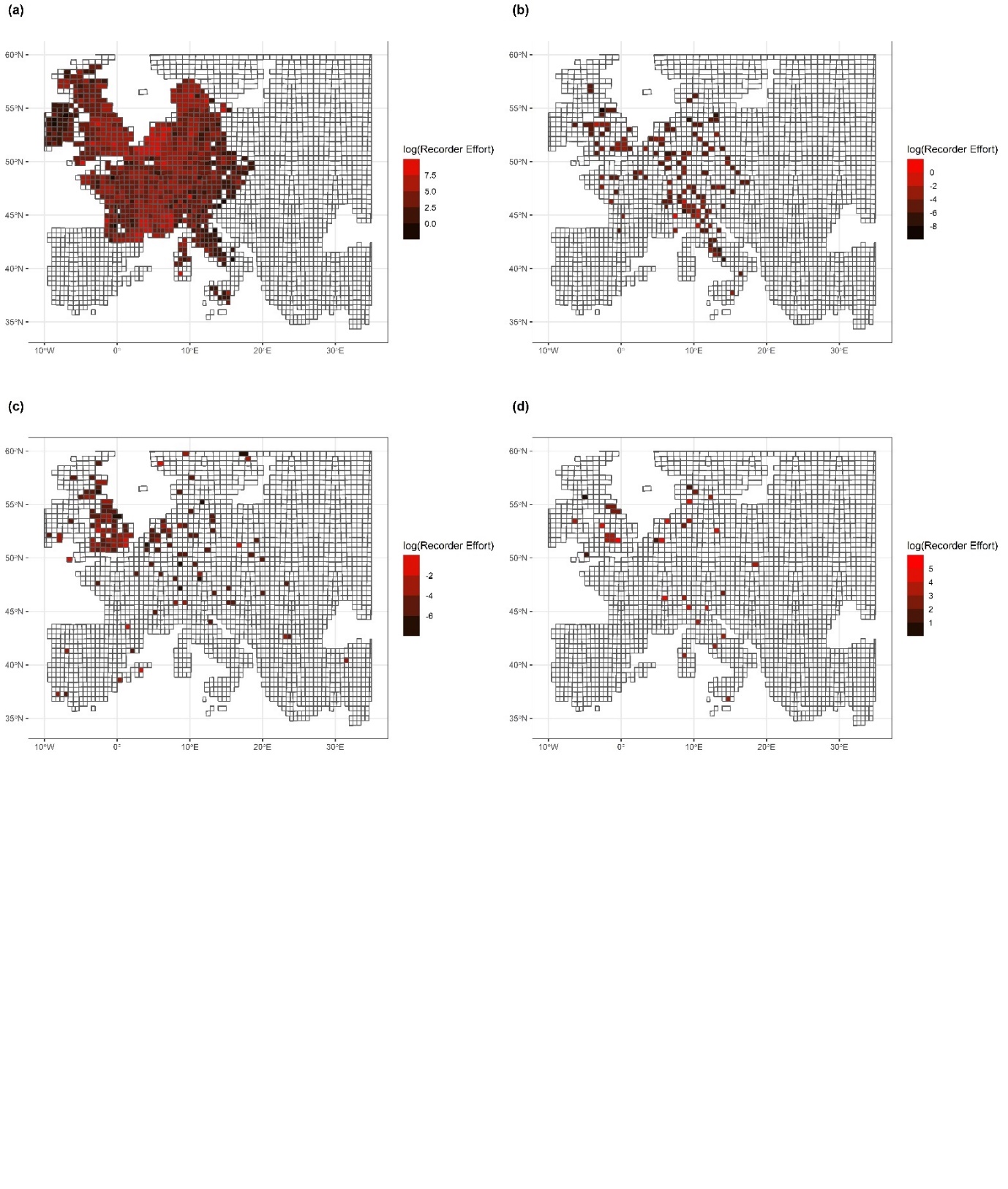


**Figure S1 | Recorder effort in 2016 from different data sources.** (a) GBIF recorder effort (including iNaturalist); (b) isolated iNaturalist recorder effort; (c) Flickr recorder effort; (d) Instagram recorder effort. Recorder effort was calculated as the abundance of blackbirds in a cell as reported by that data source divided by the actual estimated abundance according to data from the European Breeding Bird Atlas^36^. Grid size is approximately 50 km^2^, although some gird cells varied in size. Grid was supplied by European Breeding Bird Atlas.


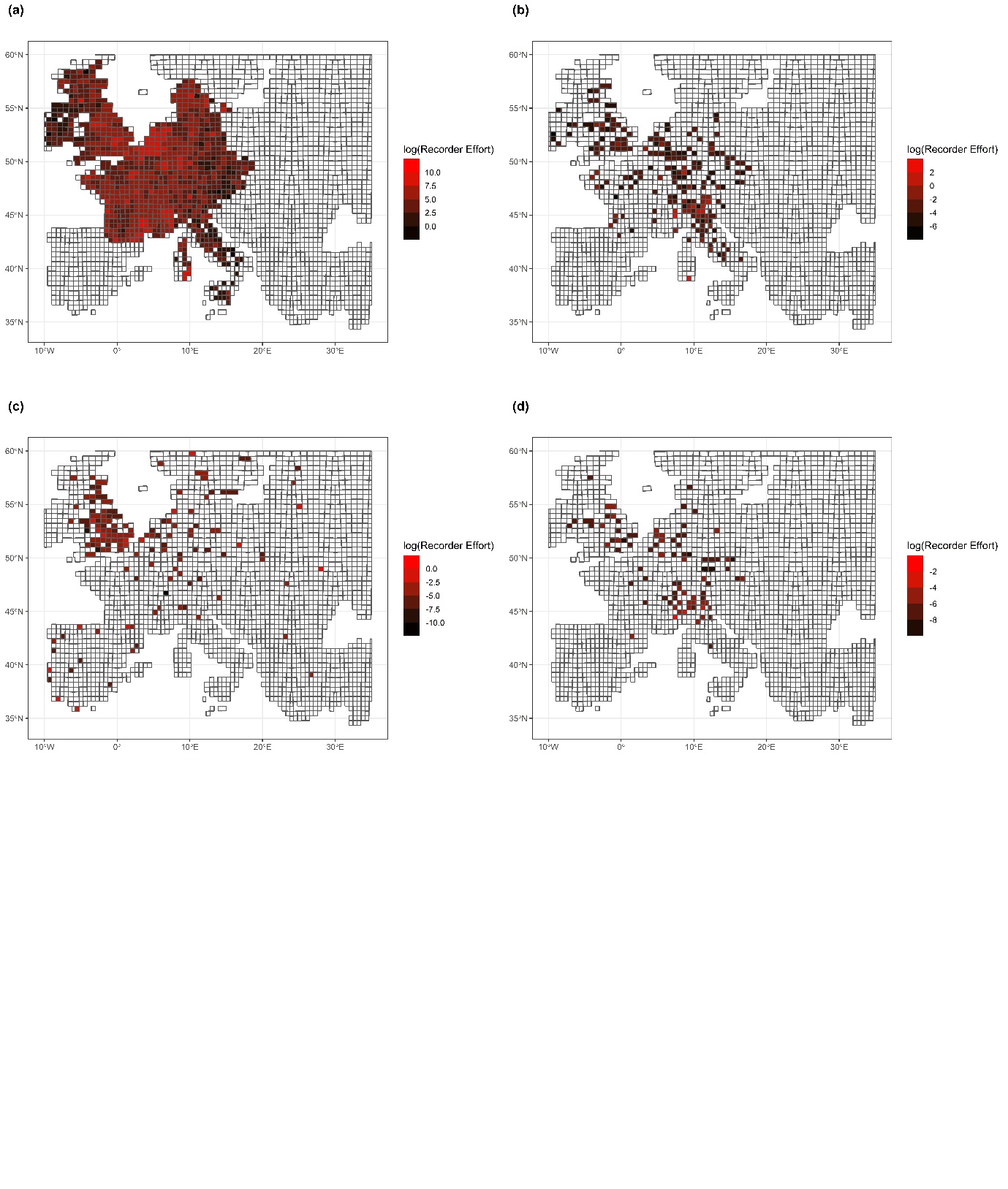


**Figure S2 | Recorder effort in 2017 from different data sources.** (a) GBIF recorder effort (including iNaturalist); (b) isolated iNaturalist recorder effort; (c) Flickr recorder effort; (d) Instagram recorder effort. Recorder effort was calculated as the abundance of blackbirds in a cell as reported by that data source divided by the actual estimated abundance according to data from the European Breeding Bird Atlas^36^. Grid size is approximately 50 km^2^, although some gird cells varied in size. Grid was supplied by European Breeding Bird Atlas.


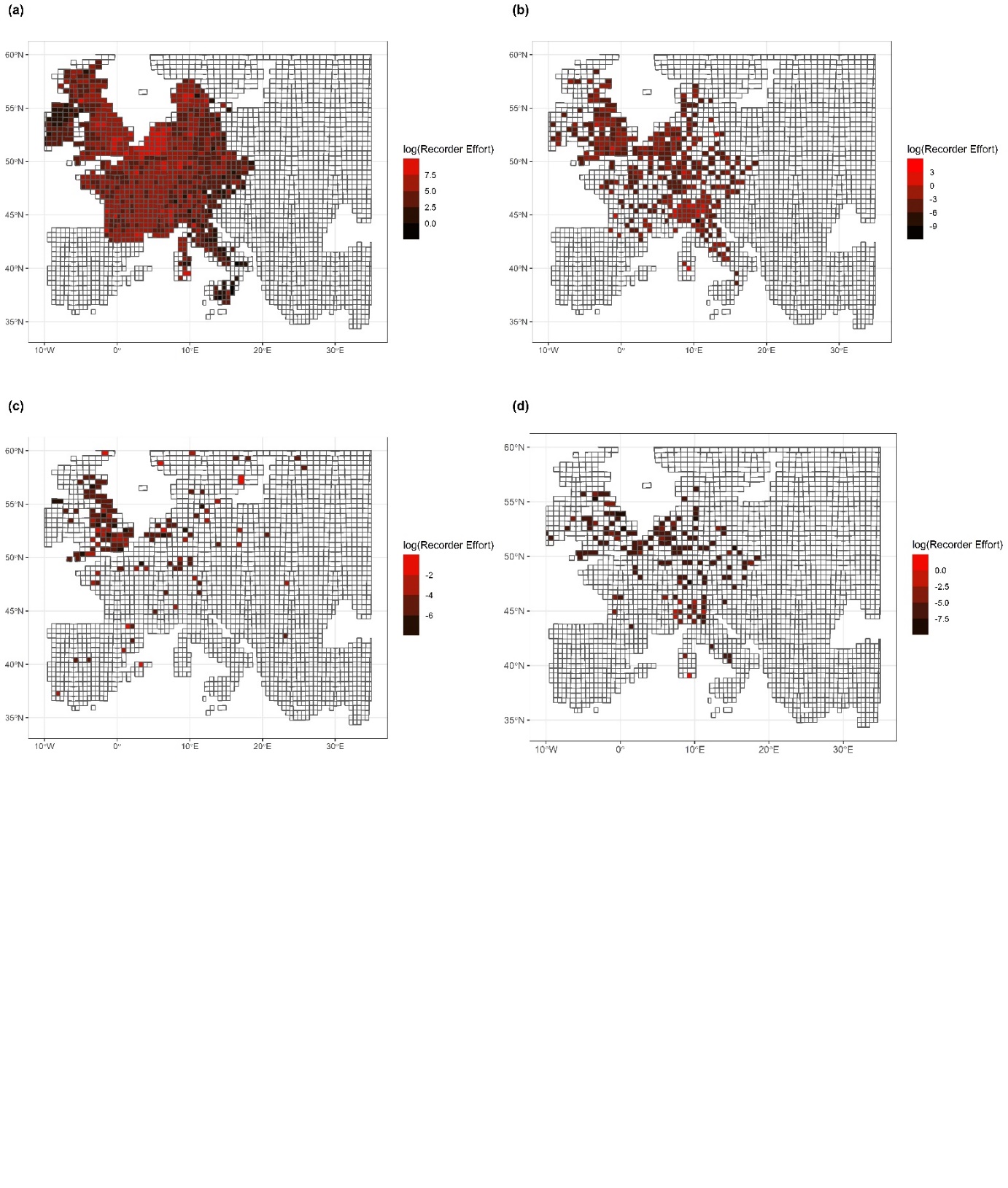


**Figure S3 | Recorder effort in 2018 from different data sources.** (a) GBIF recorder effort (including iNaturalist); (b) isolated iNaturalist recorder effort; (c) Flickr recorder effort; (d) Instagram recorder effort. Recorder effort was calculated as the abundance of blackbirds in a cell as reported by that data source divided by the actual estimated abundance according to data from the European Breeding Bird Atlas^36^. Grid size is approximately 50 km^2^, although some gird cells varied in size. Grid was supplied by European Breeding Bird Atlas.


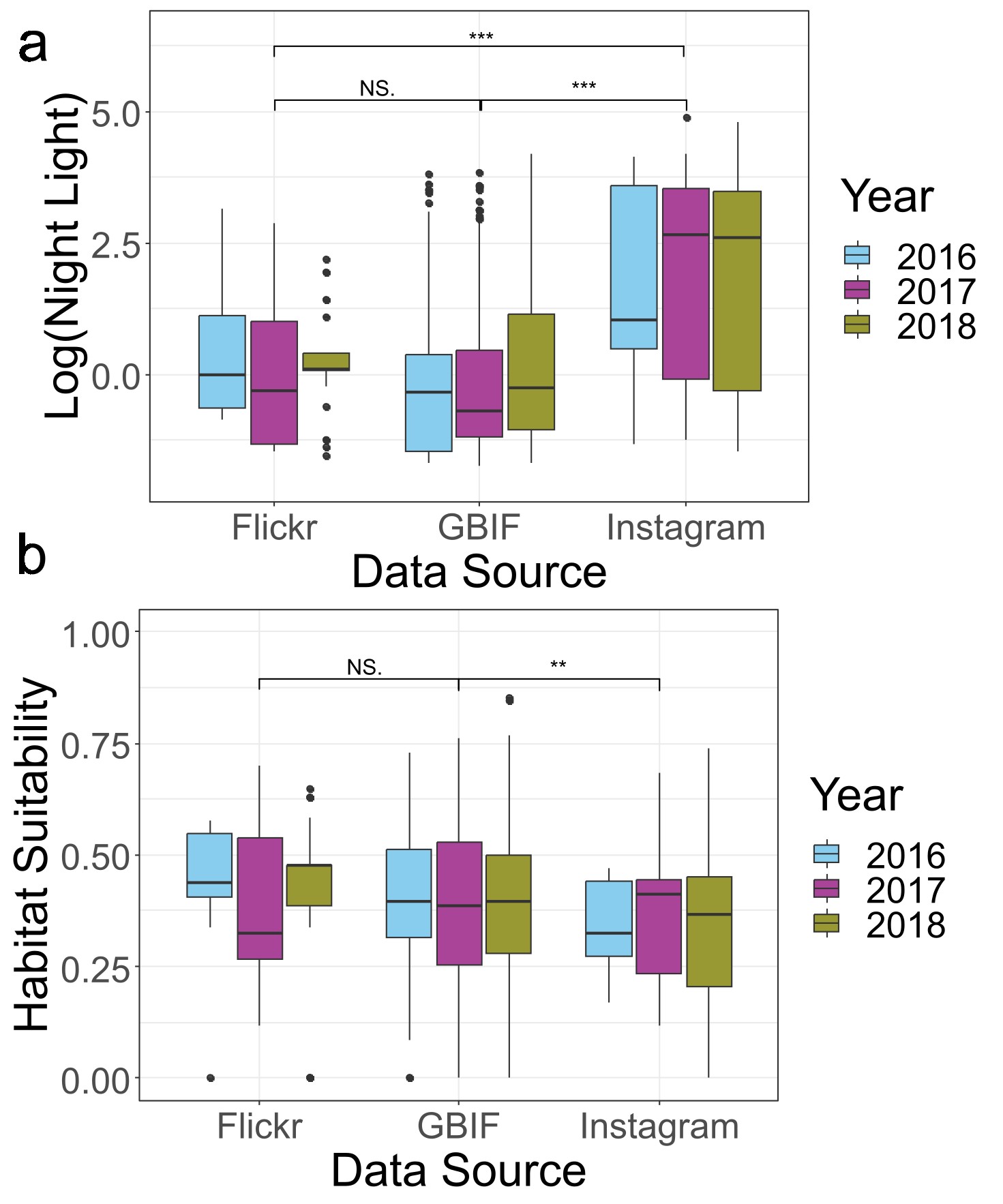


**Figure S4 | Differences in (a) night light (urbanisation) and (b) habitat suitability between different sources of data with Italy removed from the study region**. (a) and (b): horizontal black bars denote median; vertical bars denote quantiles; NS denotes no significant difference, asterisks denote statistically different variables, and quantity of asterisks denote size of *p*-value (** = *p* < 0.01).


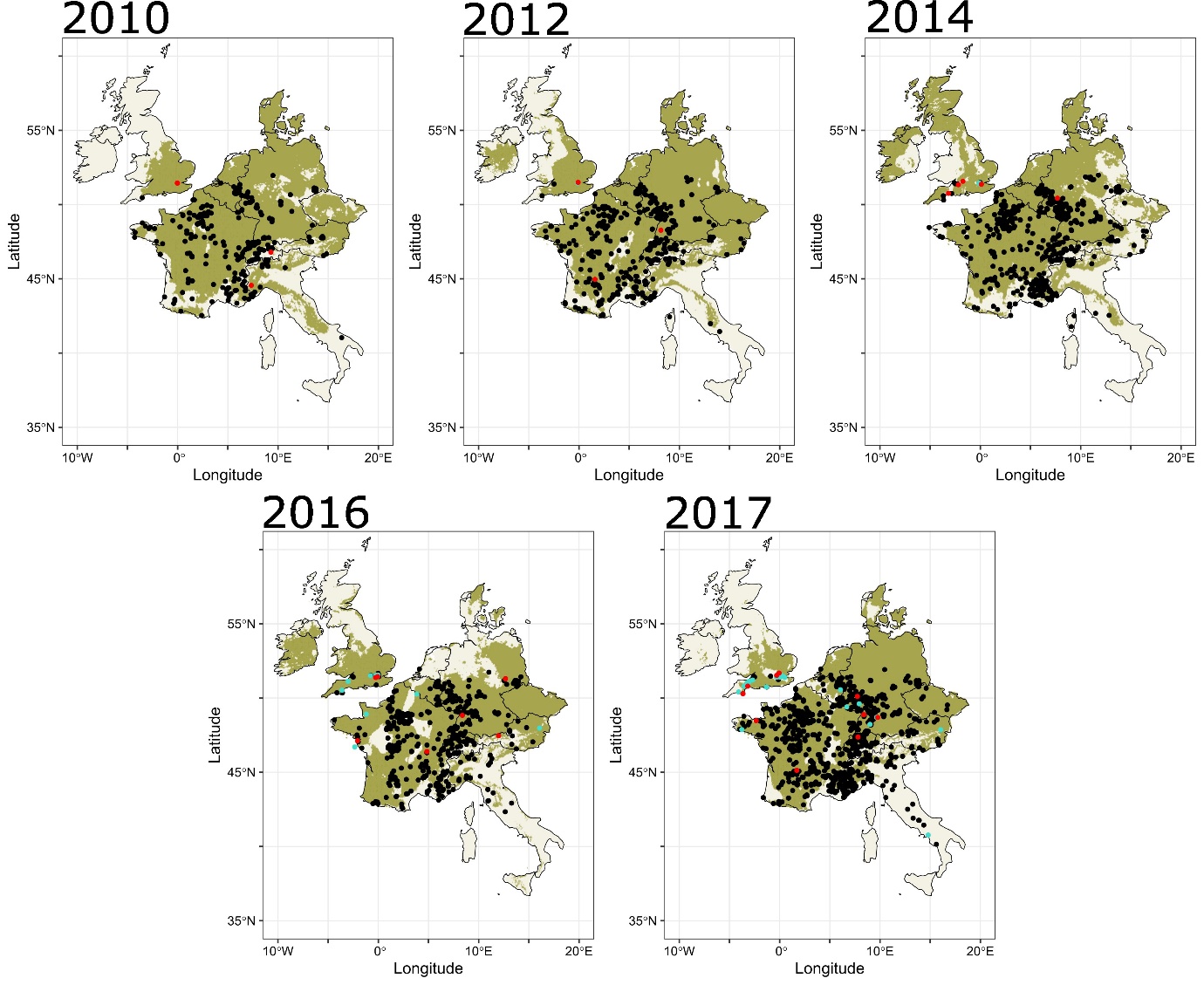


**Figure S5 | Additional HSMs for JTM across the study region not included in main text.** Green areas represent suitable habitat for JTM (sensitivity = 0.9); black points are from GBIF; red points originate from Flickr and turquoise Instagram. Habitat suitability was calculated from maximum temperature, covariance in maximum temperature, total precipitation, and covariance in total precipitation using BioClim models produced with the *dismo* package in R.


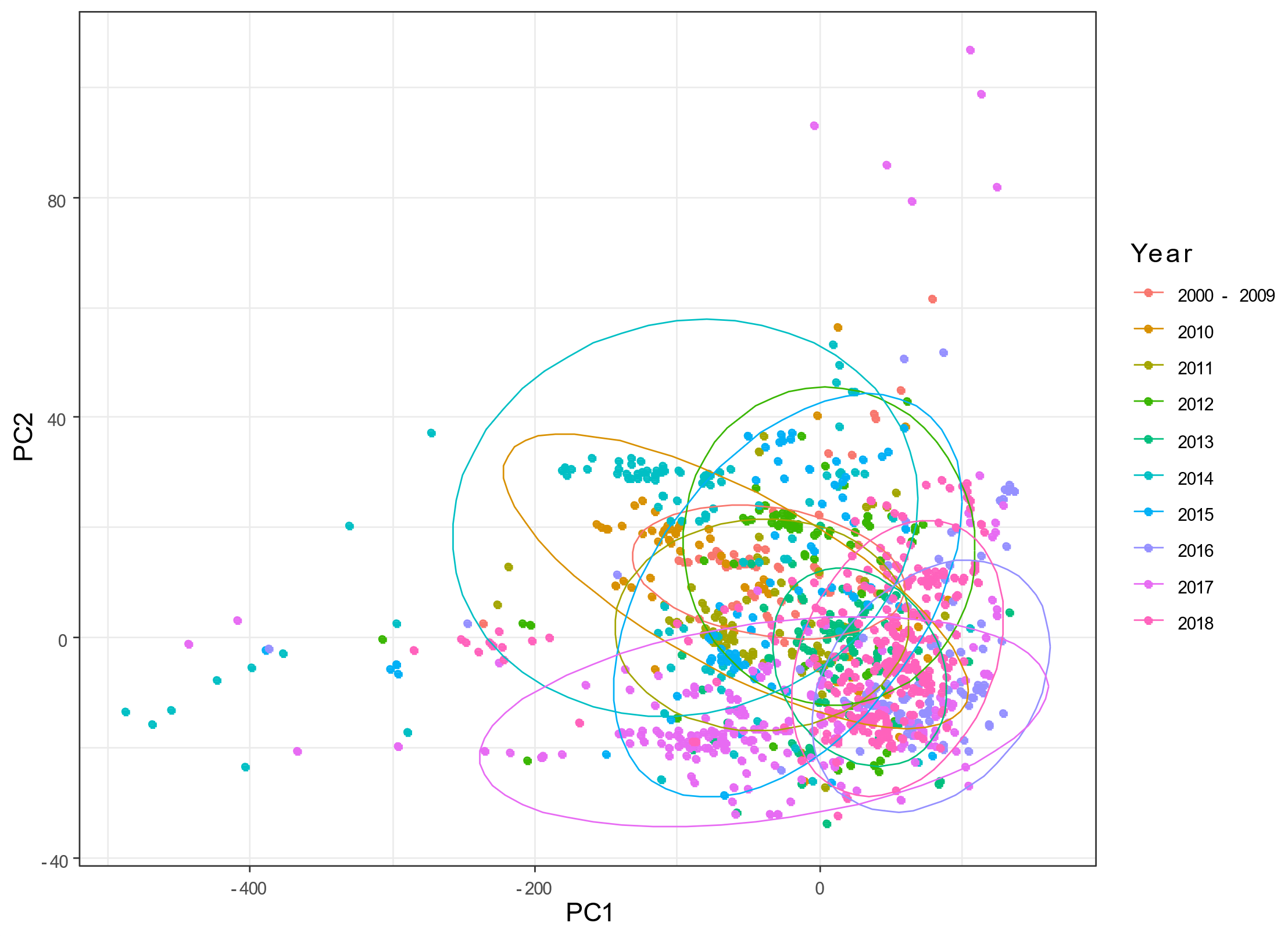


**Figure S6 | PCA** **plot containing climatic variables from all occurrences of JTM across years included in this study.** Overlap of ellipses suggests that climatic variables where JTM is found have not differed over time. Ellipses plotted using 95% confidence intervals.


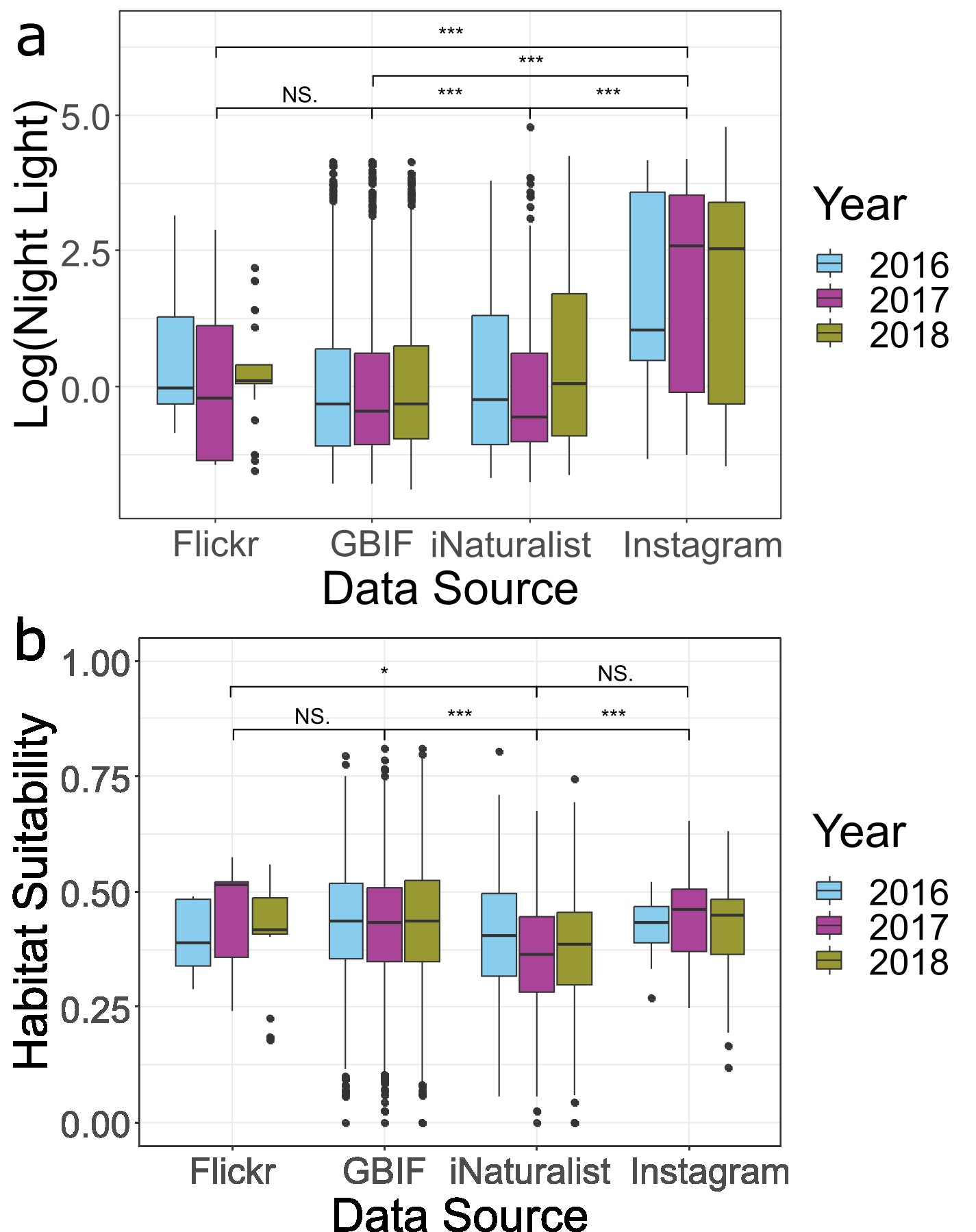


**Figure S7 | Differences in (a) night light (urbanisation) and (b) habitat suitability between different sources of occurrence data**. Data were taken from the selected study region across 2016 - 2018. (a) and (b): horizontal black bars denote median; vertical bars denote quantiles; NS denotes no significant difference, asterisks denote statistically different variables, and quantity of asterisks denote size of *p*-value (* = *p* < 0.05; *** = *p* < 0.001). Non-significant results (Table S2) not figured to aid with eligibility.


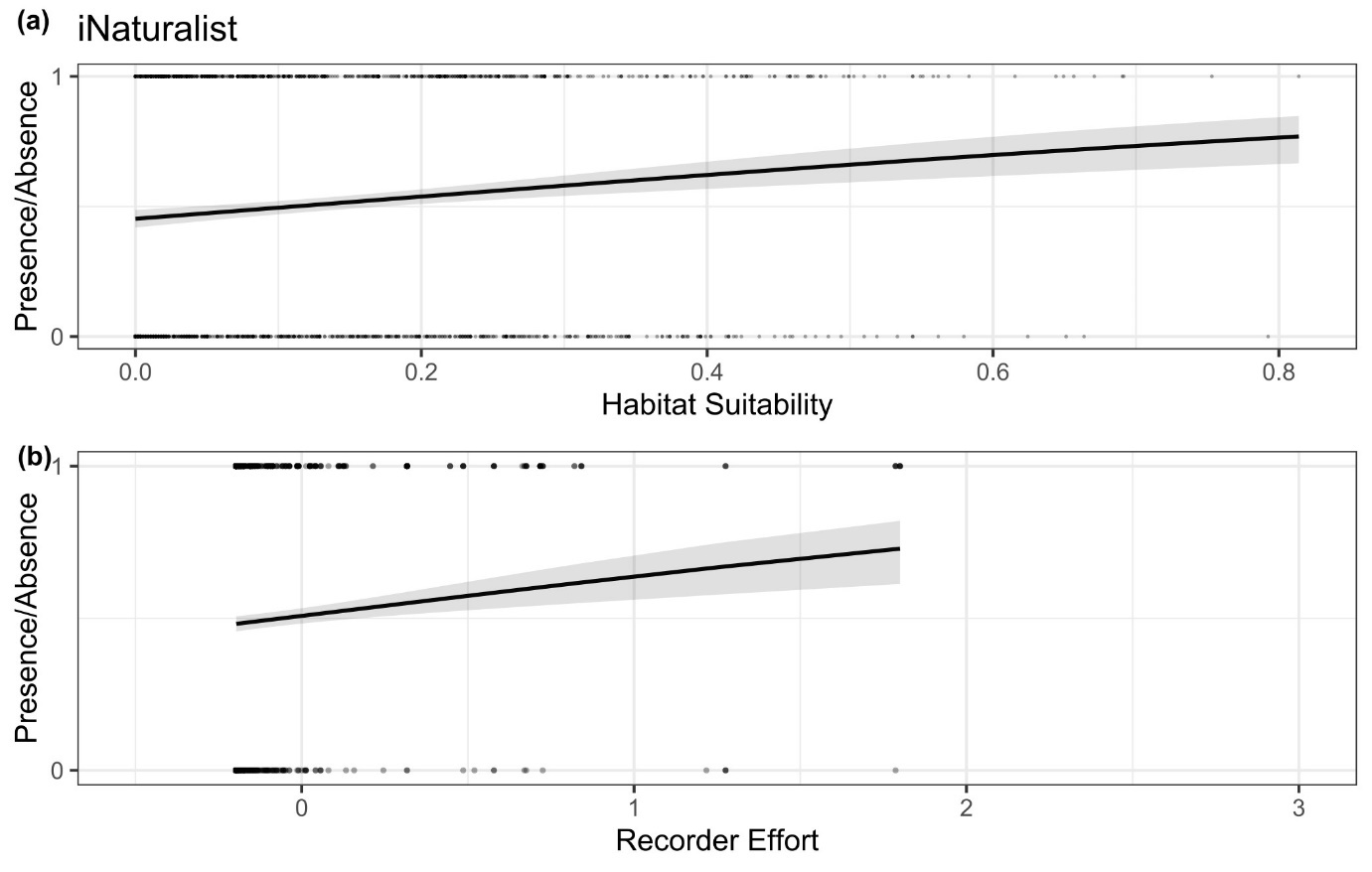


**Figure S8 | Effect of standardised values of habitat suitability (a) and recorder effort (b) on the presence of JTM for iNaturalist data.** 0 on the y axis refers to a pseudoabsence, 1 refers to presence of JTM. Grey area denotes 95% confidence interval.

**Table S1 | Summary of the search terms and processes used to collect biological records of the Eurasian blackbird across the study region. We used the species’ common name in each of the countries studied.** Note that searches on Instagram are limited to hashtags rather than caption text. Data are available at <https://figshare.com/s/94529defd9aa93d18426>, except data from GBIF (link in table).

| **Data source** | **Search terms(s)** | **Process** |
| --- | --- | --- |
| GBIF | *Turdus merula* | Downloaded from GBIF GBIF.org (02 July 2020) GBIF Occurrence Download https://doi.org/10.15468/dl.dn3vez |
| Twitter | *Turdus merula* | Manual search |
| Flickr | *Turdus merula*  Blackbird  Amsel  Merel  Merle noir  Solsort | Automatic API search using python code and then query geographical data using *FlickrAPI* package in R ^37,47^. These were checked manually to remove non-blackbird occurrences. |
| Instagram | #Turdusmerula  #Amsel  #Merel  #Morle noir  #Solsort | Manual search |

**Table S2 | Summary of Tukey’s post-hoc tests between (log) habitat suitability and urbanisation (square root night light) for four sources of JTM occurrence data.**

| **Comparison** | **Habitat suitability difference** | **Habitat suitability *p*-value** | **Urbanisation difference** | **Urbanisation *p*-value** | |
| --- | --- | --- | --- | --- | --- |
| GBIF - Flickr | 0.004 | 0.996 | - 0.278 | | 0.519 |
| iNaturalist - Flickr | -0.051 | **0.048** | 0.076 | | 0.983 |
| Instagram - Flickr | 0.003 | 0.999 | 1.541 | | **< 0.001** |
| iNaturalist - GBIF | -0.056 | **< 0.001** | 0.354 | | **< 0.001** |
| Instagram - GBIF | 0.002 | 0.999 | 1.819 | | **< 0.001** |
| Instagram - iNaturalist | 0.054 | **< 0.001** | 1.465 | | **< 0.001** |

**Table S3 | AIC scores for model selection process for recorder effort models.** (a) Models produced with GBIF data; (b) models produced with Flickr data; (c) models produced with Instagram data.

(a)

| **Habitat suitability** | **Recorder effort** | **Degrees of freedom** | **Log Likelihood** | **AICc** | **ΔAICc** |
| --- | --- | --- | --- | --- | --- |
| + | + | 3 | - 3832.320 | 7676.6 | 0.000 |
| + | - | 2 | - 3866.203 | 7736.4 | 59.8 |
| - | + | 2 | - 3972.712 | 7949.4 | 272.8 |
| - | - | 1 | - 4008.470 | 8018.9 | 342.3 |

(b)

| **Habitat suitability** | **Recorder effort** | **Degrees of freedom** | **Log Likelihood** | **AICc** | **ΔAICc** |
| --- | --- | --- | --- | --- | --- |
| - | + | 2 | -65.494 | 135.1 | 0.00 |
| - | - | 1 | -66.542 | 135.1 | 0.01 |
| + | - | 2 | -66.167 | 136.5 | 1.34 |
| + | + | 3 | -65.199 | 136.7 | 1.54 |

(c)

| **Habitat suitability** | **Recorder effort** | **Degrees of freedom** | **Log Likelihood** | **AICc** | | **ΔAICc** |
| --- | --- | --- | --- | --- | --- | --- |
| - | + | 2 | -171.187 | 346.4 | | 0.000 |
| + | + | 3 | -171.124 | 348.3 | | 1.90 |
| - | - | 1 | -176.751 | 355.5 | | 9.08 |
| + | - | 2 | -176.558 | 357.2 | 10.72 | |

**Table S4 | AIC scores for model selection process for recorder effort models where iNaturalist was separated from GBIF data.** (a) Models produced with GBIF (minus iNaturalist) data; (b) models produced with iNaturalist data.

1. GBIF

| **Habitat suitability** | **Recorder effort** | **Degrees of freedom** | **Log Likelihood** | **AICc** | **ΔAICc** |
| --- | --- | --- | --- | --- | --- |
| + | + | 3 | - 3821.456 | 7648.9 | 0.00 |
| + | - | 2 | - 3848.052 | 7700.1 | 51.19 |
| - | + | 2 | - 3936.458 | 7876.9 | 228.00 |
| - | - | 1 | - 3968.268 | 7938.5 | 289.62 |

1. iNaturalist

| **Habitat suitability** | **Recorder effort** | **Degrees of freedom** | **Log Likelihood** | **AICc** | | **ΔAICc** |
| --- | --- | --- | --- | --- | --- | --- |
| + | + | 3 | -1155.861 | 2293.401 | | 0.00 |
| - | + | 2 | -1166.671 | 2301.561 | | 19.61 |
| + | - | 2 | -1174.404 | 2356.029 | | 35.08 |
| - | - | 1 | -1185.281 | 2364.245 | 54.83 | |
